# *Culex modestus:* the overlooked mosquito vector

**DOI:** 10.1186/s13071-023-05997-6

**Published:** 2023-10-20

**Authors:** Alina Soto, Leen Delang

**Affiliations:** Department of Microbiology, Immunology and Transplantation, Rega Institute for Medical Research, Laboratory of Virology and Chemotherapy, KU Leuven, Leuven, Belgium

**Keywords:** *Culex modestus*, Mosquito, Vector, Arbovirus, Avian malaria, Canine filariasis

## Abstract

*Culex *(*Barraudius*) *modestus* (Ficalbi 1889) are found in temperate regions across Europe, Asia, and Northern Africa. These mosquitoes thrive during the summer and prefer to breed in permanent vegetative habitats such as rice paddies and marshes. *Culex modestus* feed on a wide range of bird species but are highly attracted to humans, which makes them a potential ‘bridge’ vector for enzootic pathogens. There is compelling evidence that *Culex modestus* is an efficient vector for West Nile virus, potentially capable of causing epidemics in humans and other mammals. This species is also a likely vector for Usutu virus, avian malaria (*Plasmodium* spp.), and parasitic heartworms (*Dirofilaria* spp.). *Culex modestus* can be morphologically identified at the larval and adult stages, and a distinctive phenotype of this species is their ability to overwinter. Despite the widespread establishment of this mosquito species and their role as vectors for human pathogens, we lack sufficient knowledge on this species to implement and evaluate targeted vector control measures. Since *Culex modestus* can be considered a potential public health threat, there is a need for a better understanding of this mosquito species.

**Graphical Abstract:**

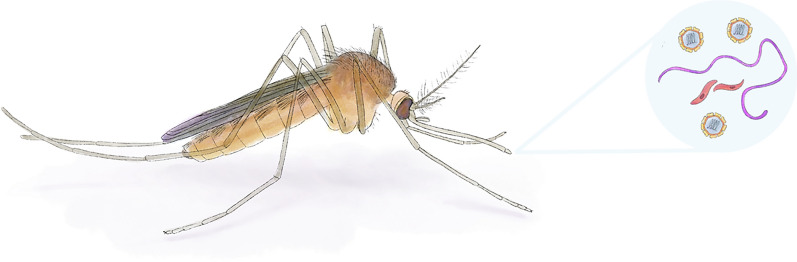

**Supplementary Information:**

The online version contains supplementary material available at 10.1186/s13071-023-05997-6.

## Background

*Culex* (*Cx.*) mosquitoes of the *Culicidae* family are important global vectors for human and animal pathogens, including arthropod-borne viruses (arboviruses) and eukaryotic parasites. In Europe, the northern house mosquito *Cx. pipiens* sensu lato has been traditionally considered the primary mosquito vector responsible for pathogen transmission. They are a known vector for West Nile virus, Japanese encephalitis virus, Rift Valley fever virus, Sindbis virus, Tahyna virus, dirofilarial worms, and avian malaria [1]. Yet, a lesser known member of the *Culex* genus—*Cx. (Barraudius) modestus*—may play an important role in pathogen transmission as well. Little is known about this vector, but evidence suggests that *Cx. modestus* is more anthropophilic (human-biting) and potentially more competent as a West Nile virus vector than *Cx. pipiens* s.l. mosquitoes. In this article we present a review of the literature, including what is known, and what is unknown, about the important but overlooked mosquito vector *Cx. modestus*.

### Ecology

#### Geographic distribution

**Fig. 1:**
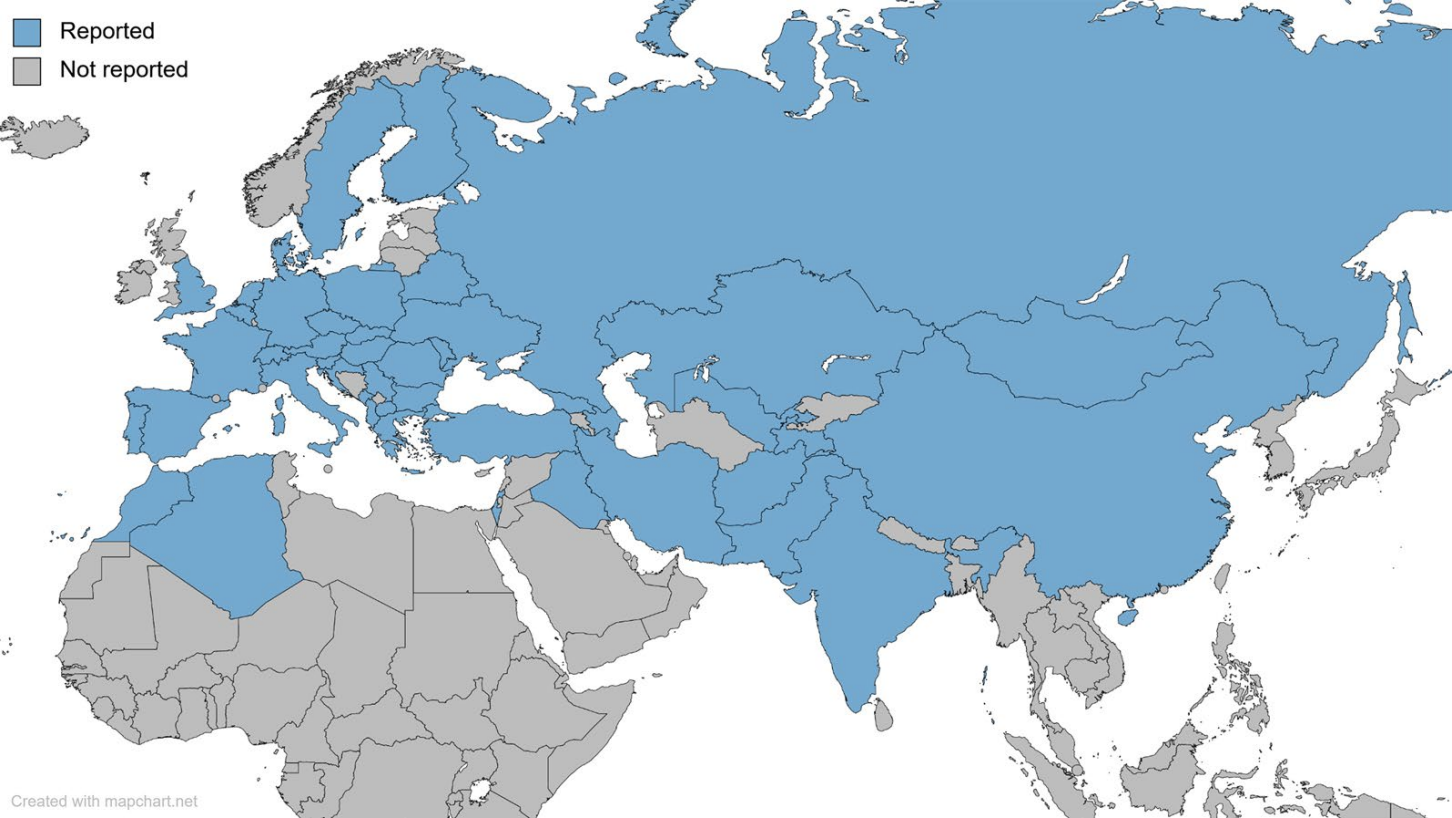
Map of *Culex modestus* distribution (as of August 2023). Countries highlighted in blue represent those with at least one report of *Cx. modestus*, whereas those in grey indicate that *Cx. modestus* are absent or have not yet been reported. Sources used in the creation of this map can be found in the supplementary material (Additional file 2: Table S1). Created with Mapchart.net

*Culex modestus* was first identified in 1889 in Ravenna, Italy [2], and has since been discovered across a wide landscape of countries in Europe, northern Africa, and Asia (Fig. 1). The current distribution of native *Cx. modestus* mosquitoes at the regional level in Europe can be found on the European Centre for Disease Prevention and Control (ECDC) website, where they provide regular updates on the distribution of native and invasive mosquito species [3].

The *Cx. modestus* genotype can be divided into lineages I and II based on haplotypes in the mitochondrial cytochrome oxidase 1 gene (COX1) [4, 5]. Lineage I has been found in Spain and Portugal, lineage II was found in Germany, Sweden, and the UK, and both lineages were found in Belgium, France, Serbia, and Denmark*.* A haplotype analysis suggested that *Cx. modestus* from the UK and Germany share a common origin from France, whilst another part of the UK population may have been derived from Serbia [5]. In Belgium, *Cx. modestus* were likely derived from the UK and Germany, possibly the result of several independent introduction events [4]. Phylogeny on the basis of rRNA intergenic spacers between the 28S rRNA gene (3’ end) and 18S rRNA gene (5’ end) on chromosome I revealed that *Cx. modestus* is highly evolutionarily distant from *Cx. pipiens pipiens*, *Cx. pipiens molestus*, and *Cx. torrentium* [6].

It would be interesting to investigate the origin of *Cx. modestus* and how they have spread inter- and cross-continentally. A small number of *Cx. modestus* were found breeding in used tires during a large national survey in Spain [7], alerting to a potential mode of dispersal to new areas. In a mosquito survey of international ships arriving to Hebei Province in China, more than half of the vessels were found with mosquitoes onboard [8]. Almost all the mosquitoes on these ships were *Culex* (97%), and *Cx. modestus* and *Cx. tritaeniorhynchus* were the dominant species. Sea travel could therefore be a likely route of migration for these species. Given the extensive establishment of *Cx. modestus* across Eurasia and northern Africa, potential introduction to new areas by global transport, and how the effects of climate change are expected to develop more suitable mosquito climates and habitats across the globe, this species will probably appear in new areas.

#### Habitat

In contrast to *Cx. pipiens* s.l., which can be found scattered across a wide range of ecological habitats, *Cx. modestus* are generally restricted to specific rural and agricultural breeding sites [9]. Their larvae are found in mostly permanent water bodies including rice fields [10–12], reedbeds [11, 13, 14], fish ponds [9, 15–17], wetlands [9, 11, 18], marshes [11, 19, 20], and woodlands [13] or deciduous forests [14]. They have been found co-habitating with other mosquito species, mostly those of the Anopheline and Culicine genera (Table 1).

**Table 1:** Mosquito species found to co-habitate with *Culex modestus* larvae

Genus	Species	Country	Habitat	Source
*Aedes*	*Ae. (Ochlerotatus) caspius*	Iran	Unspecified	[21]
*Anopheles*	*An. claviger*	Algeria	Peri-urban habitat	[22]
	*An. hyrcanus*	Unspecified	Rice fields	[10]
	*An. labranchiae*	Algeria	Peri-urban habitat	[22]
	*An. maculipennis* s.l	Moldova	Unspecified	[23]
	*An. melanoon*	France	Rice fields and reed beds	[11]
	*An. sacharovi*	Unspecified	Rice fields	[10]
*Culex*	*Cx. longiareolata*	Algeria	Peri-urban habitat	[22]
	*Cx. pipiens* s.l	Algeria	Peri-urban habitat	[22]
		Belgium	Peri-urban habitat	[4]
		Iran	Unspecified	[21]
	*Cx. territans*	Moldova	Unspecified	[23]
	*Cx. theileri*	Algeria Iran	Peri-urban habitat Unspecified	[21, 22]
		Unspecified	Unspecified	[10]
		Moldova	Unspecified	[23]
	*Cx. torrentium*	Moldova	Unspecified	[23]
*Culiseta*	*Cs. annulata*	Moldova	Unspecified	[23]
	*Cs. subochrea*	Iran	Unspecified	[21]
*Uranotaenia*	*Ur. unguiculata*	Unspecified	Unspecified	[10]
		Moldova	Unspecified	[23]

Specific details regarding the ecological and climatic factors that influence *Cx. modestus* breeding are based on a limited number of studies. A cross-sectional survey conducted across 46 locations in northwestern Iran described the larval habitats of *Cx. modestus* in detail [24]. Most *Cx. modestus* were found in natural habitats (85%) as opposed to artificial ones (15%), in water bodies that were permanent (90%) rather than temporary (10%). Most were found in water that was slow running (73%) as opposed to stagnant (27%) and transparent (98%) versus opaque (2%). Most habitats contained vegetation (85%) as opposed to no vegetation (15%), with a bed type not based on clay or sand (55%). Most larvae were found in semi-shade (60%) compared to sunny (4%) or shade (36%). Similarly, *Cx. modestus* in Algeria could only be found at peri-urban as opposed to urban habitats [22]. The peri-urban sites were described as having open, permanent, stagnant, and clean water with an average pH of 7.7. In contrast, a different larval sampling survey in Iran found more *Cx. modestus* in urban than rural areas; however, only a small number of *Cx. modestus* was recovered and represented only a small proportion of the overall mosquito population (2.4%, *n* = 33) [25]. The ability to grow in urban settings suggests that *Cx. modestus* are adaptable to diverse environments.

In a random sampling of adult mosquitoes at a nature reserve in Spain, the abundance of *Cx. modestus* was positively associated with inundation area (i.e. water surface) and hydroperiod (i.e. water permanence) [20]. The *Cx. modestus* displayed a preference for hydroperiods of > 150–200 days/year. In a 7-year surveillance study of adult mosquitoes in the Piedmont Region of Italy, proximity to rice fields was positively correlated to population clusters and abundance of *Cx. modestus* [26]. Elevation and distance from breeding sites, on the other hand, were negatively associated with abundance. When comparing two mosquito seasons—a wetter season with a colder average temperature (2002: 20.5 °C, 512 mm) vs. a drier season with a warmer average temperature (2003: 23 °C, 122 mm)—more *Cx. modestus* were captured during the wetter and colder season [26], demonstrating the importance of water surface area for *Cx. modestus* breeding.

The abundance of *Cx. modestus* was shown to be affected by agricultural changes. In fact, the rapid proliferation and subsequent near elimination of *Cx. modestus* are well described in the Camargue region of France [27]. *Culex modestus* was rarely reported in the Camargue until a massive upscale in rice cultivation following World War II. The population of *Cx. modestus* as well as *An. hyrcanus* became widespread and abundant throughout the region where they were considered major pests and nuisance biters. In that period, increases in mosquito density could thus be attributed to changes in paddy surface area, which suggests that reed marshes alone cannot sustain populations of *Cx. modestus* to the same extent as rice paddies. In later years, rice cultivation in France started to decrease and insecticide use in remaining paddies was implemented to combat pests. Consequently, the mosquito populations in these fields declined. Since the start of the twenty-first century, insecticide use has been slowly replaced by more sustainable pest control methods, resulting in a gradual increase in mosquito populations once again.

### Biology

#### Lifecycle

Populations of *Cx. modestus* bloom during the warm summer months. Most studies that measured levels of *Cx. modestus* across different periods captured larger numbers in the months of July and August [19, 26, 28–35]. One study in Romania captured more *Cx. modestus* in late August or early September [36], because, interestingly, while more *Cx. pipiens* s.l. were captured over the summer months, towards the end of the season the proportion of *Cx. modestus* exceeded that of *Cx. pipiens* s.l. mosquitoes. In France, more *Cx. modestus* were captured in June or July [37]. One sampling study from Spain found a significantly higher proportion captured in marshlands in June (98%) than in any other habitat or time between March and November [20]. Likewise, a study from Moldova also captured a higher number in June (49.4%) than any other time between May and September [38]. A sampling study in Algeria found *Cx. modestus* from May to October with temperatures ranging from 17.9 at night to 31.4 °C during the day and rainfall between 0 and 66 mm [22].

Little is known about the life cycle of *Cx. modestus* and what sets it apart from other members of the *Culex* genus*. Culex modestus* has been reported to overwinter (diapause) [13, 39], a phenomenon characterized by the ability of female mosquitoes to ‘hibernate’ during the cold months of the year. Like *Cx. pipiens pipiens* and *Culex tarsalis*, female *Cx. modestus* enter diapause when temperatures begin to fall after the summer [40], which encourages female mosquitoes to fly to secluded shelters such as caves or bunkers. When outdoor temperatures begin to rise again, females exit diapause and seek a blood meal to produce a batch of eggs. This adaptation to the cold raises concern over potential arbovirus carry-over between transmission seasons [41].

The potential for *Cx. modestus* to produce a first batch of eggs without a prior blood meal (autogeny) was briefly mentioned in one research article in which larvae were captured in the Padurea Domneasca reserve in Moldova and reared to adulthood in the laboratory [23]. The researchers observed that female adults deposited a first batch of viable eggs after sugar feeding without a prior blood meal, and the same was observed in the next generation. This is currently the only report of autogeny in *Cx. modestus*.

Three research groups have reported the rearing of *Cx. modestus* in the laboratory. A colony from Lattes, France, was maintained at 26 ± 1 °C with > 50% RH and a 16-h photoperiod with 1.5 h of simulated dawn/dusk crepuscular periods [42, 43]. Larvae were reared in pans containing tap water and yeast tablets [43], while adults were provided with 10% sugar solution and heparinized rabbit blood for egg production [42, 43]. A laboratory colony of *Cx. modestus* (Beijing strain) in China was maintained at 26 ± 1 °C and 75 ± 5% RH under a 14-h photoperiod [44]. In a study from Greece that used F_0_ adults reared from larvae, the larvae were captured and consequently reared in the laboratory in pans (53.3 × 40.6 cm) filled with deionized water and aerated with aquarium pumps [45]. Larvae were fed a mixture of liver and yeast powder medium (2:3 ratio) under natural light-dark conditions at 25 °C and > 60% RH, and adult mosquitoes were provided with 10% sugar solution.

#### Host preference

*Culex modestus* are known to feed on birds and mammals interchangeably, which could make this species a good ‘bridge’ vector for enzootic pathogens such as West Nile virus. While it has been suggested that *Cx. modestus* displays a preference for feeding on birds (ornithophily) [46], certain data from literature show a strong disposition for human biting. A study evaluating human biting willingness in mosquitoes found that *Cx. modestus* and other human-biting (anthropophilic) species such as *Anopheles plumbeus*, *Aedes* *vexans*, *Ae. (Ochlerotatus) sticticus*, and *Cx. pipiens molestus* were positively correlated to human biting (> 0.7 human biting willingness), whereas species such as *Cx. pipiens pipiens* and *Culex territans* were negatively correlated (< 0.1 human biting willingness) [47]. The relative biting risk index (RBRI) for *Cx. modestus* (0.07 RBRI) was higher than that of 38 out of 42 species included in the study but lower than for *Ae. vexans* (> 0.4 RBRI), *Aedes (Ochlerotatus) annulipes* (0.25 RBRI), and *Ae. (Ochlerotatus) sticticus* (> 0.1 RBRI) [47]. Another study observed that *Cx. modestus* were more attracted to humans than to birds, whereas *Cx. pipiens* s.l. were more attracted to birds [48]. Recent evidence therefore suggests that *Cx. modestus* displays more anthropophilic or mammalophilic rather than ornithophilic feeding behaviour.

Known hosts of *Cx. modestus* are based on a limited number of studies (Table 2). *Culex modestus* has been shown to feed on freshwater dwellers such as ducks, geese, and herons; mammals and farmland animals such as dogs, chickens, cattle, and horses; and even birds of prey (i.e. Western Marsh harrier) and reptiles. Given the natural aquatic habitats of *Cx. modestus* in areas such as rice paddies and marshes, it is fitting that they feed on animals from the same habitats, such as wild aquatic birds or rural farmland animals. It is interesting that some studies have found this species to be more attracted to humans than to birds, but blood-meal host identification on field-captured mosquitoes found more mosquitoes that fed on birds than mammals. This suggests that the feeding preference of *Cx. modestus* depends on host abundance and availability in their current habitats.

**Table 2:** Host sources of *Culex modestus* based on blood-meal identification or host-feeding studies

Class	Scientific name	Common name	Country	Source
Bird (Aves)	*Anas acuta*	Northern pintail	Spain	[100]
	*Anas platyrhynchos*	Mallard	Czech Republic	[9]
			Spain	[46, 100]
	*Anas strepera*	Gadwall	Czech Republic	[9]
			Spain	[100]
	*Anas* sp.	Duck (unspecified)	China	[53]
			France	[30]
			Spain	[101]
	*Anser anser*	Greylag goose	Czech Republic	[9]
			Spain	[46, 100]
	*Ardea cinerea*	Grey heron	Spain	[100]
	*Branta canadensis*	Canada goose	Spain	[100]
	*Chen caerulescens*	Snow goose	Spain	[100]
	*Circus aeruginosus*	Western Marsh harrier	Czech Republic	[9]
	*Cygnus olor*	Mute swan	United Kingdom	[28]
	*Egretta garzetta*	Little egret	Spain	[100]
	*Emberiza citrinella*	Yellowhammer	Czech Republic	[9]
	*Erithacus rubecula*	European robin	Czech Republic	[9]
	*Falco vespertinus*	Red-footed falcon	Hungary	[96]
	*Fringilla coelebs*	Eurasian chaffinch	Czech Republic	[9]
	*Gallus gallus*	Red junglefowl	Spain	[46, 100]
	*Gallus* sp.	Chicken (unspecified)	Russia	[87]
	*Hirundo rustica*	Barn swallow	Czech Republic	[9]
			United Kingdom	[28]
	*Muscicapa striata*	Spotted flycatcher	Czech Republic	[9]
	*Nycticorax nycticorax*	Black-crowned night heron	Czech Republic	[9]
	*Parus caeruleus*	Blue tit	Czech Republic	[9]
	*Parus major*	Great tit	Czech Republic	[9]
	*Passer domesticus*	House sparrow	Czech Republic	[9]
	*Sturnus vulgaris*	Common starling	Czech Republic	[9]
	*Tadorna ferruginea*	Ruddy shelduck	Spain	[100]
	*Tadorna tadorna*	Common shelduck	Spain	[100]
	*Turdus merula*	Eurasian blackbird	Czech Republic	[9]
	Unspecified	Unspecified	Spain	[34, 46]
Mammal (Mammalia)	*Bos taurus*	Cow	China	[33]
			Russia	[87]
	*Canis familiaris*	Dog	Italy	[91]
			Russia	[87]
	*Equus caballus*	Horse	France	[30]
			Russia	[87]
	*Homo sapiens*	Human	Russia	[87]
			UK	[51]
	*Sus domesticus*	Pig	China	[33]
	Unspecified	Unspecified	Spain	[34, 46]
Reptile (Reptilia)	Unspecified	Unspecified	Spain	[34]

#### Host-seeking behaviour

Understanding the host-seeking behaviour of mosquitoes can help to elucidate their vectorial capacity and determine potential target areas for vector and pest control. *Culex modestus* are nuisance biters described as having painful bites [10, 49, 50]. The hourly human biting rate of *Cx. modestus* captured in England was found to be 2.5 with a range of 0–55 [51], but most likely the daily bites and frequency of feeding will depend on the local mosquito abundance and climate. *Culex modestus* are nocturnal feeders, but different studies have reported differences in peak biting activity. One research group observed different host-seeking activities depending on the mode of trapping [32]. With CO_2_-baited traps, the host-seeking pattern was unimodal with peak trapping in the evening (87.0%) and to a lesser extent during the night (12.2%) or early morning (0.7%). With human landing catches (HLCs), host-seeking activity was bimodal with most adult mosquitoes still captured in the evening (84.9%) but a non-negligible proportion captured in the morning (15.1%). The same study found that the timing of the sunset as well as relative humidity (RH) influenced the initiation of flight and peak flight activity in this species. In another study, the biting activity of *Cx. modestus* ranged from – 0.5 to + 2 h from sunset, with peak biting at + 1 h [51]. A different study observed that *Cx. modestus* fed exclusively at night with peak activity from 22:00 to 00:00, but the timing in relation to the sunset was not reported [52]. Finally, one article reported a higher number of *Cx. modestus* captured on average at dusk (21:00 to 23:00) than at dawn (05:00 to 07:00), but the difference in mean number captured per night was small (*n* =  ~ 180 at dusk, *n* =  ~ 120 at dawn) [53].

Limited evidence on the flight range of *Cx. modestus* suggests that they have a restricted travel radius. Compared to *Cx. pipiens* s.l., *Cx. modestus* was less likely to stray from the shorelines of breeding sites [9]. The radial distance of active dispersion around an emergence site for *Cx. modestus* was estimated to be 700 m, shorter than that of *An. melanoon* (1000 m) which co-habitated in the same breeding sites [11]. A trapping study that captured mosquitoes using traps placed at different distances from the ground measured a mean flying height of 2.26 m (± 0.57) for *Cx. modestus*, which was lower than that of *Cx. pipiens* s.l. (2.66 m ± 0.90) but higher than for *Coquillettidia richiardii* (2.00 m ± 1.00), *Aedes detritus* (1.98 m ± 0.71), and *Ae. caspius* (1.74 m ± 0.24) [54]. A different study observed that more *Cx. modestus* were captured in traps placed 5 m above the ground than at 1 or 3 m, suggesting that this altitude reflected the ornithophilic behaviour of the species [53].

Observations of *Cx. modestus* in Volgograd, Russia, found that they could feed interchangeably indoors (endophagy) and outdoors (exophagy) [48]. *Culex modestus* fed indoors more frequently than *Cx. pipiens* s.l., and they fed more often on humans and other mammals both indoors and outdoors than *Cx. pipiens* s.l. mosquitoes. As the *Cx. modestus* in this study were found in both rural and urban areas, more research is needed to understand their feeding behaviour across different habitats in other locations.

#### Microbiome

Unravelling the microbiome of mosquito vectors can help to identify potential targets for control. A prime example of a microbe used in the biological control of mosquitoes is *Wolbachia pipientis*, an intracellular bacterium which has been successfully used as an antiviral strategy against dengue virus in endemic areas [55]. While *Wolbachia* is known to persistently infect *Culex* spp. [56], particularly *Cx. pipiens* s.l., which has a high prevalence of *Wolbachia*, current evidence suggests that most *Cx. modestus* do not carry this endosymbiont [57, 58]. Mosquitoes from Eastern Europe (7% prevalence rate) [58] and Italy (unknown prevalence rate) [57] have been found infected with *Wolbachia*, while one study with a low sample size (*n* = 11) did not find any *Wolbachia*-infected *Cx. modestus* from Belgium [59]. It would be interesting to investigate whether *Wolbachia* can modify arbovirus infection in *Cx. modestus*, as it has been shown to do for other species [60].

Viral metagenomic analyses of field-captured mosquitoes have identified insect-specific viruses (ISVs) belonging to *Cx. modestus* captured from Belgium [4] and eastern Macedonia and northern Greece [61] (Table 3). The latter identified several core ISVs that overlapped between *Cx. modestus* and *Cx. theileri*, *An. melanoon*, and *Uranotaenia unguiculata*, which have been found to co-habitate together. The potential role of these ISVs on the life history, survival, and vector competence of *Cx. modestus* mosquitoes is not known.

**Table 3:** Insect-specific viruses found in *Culex modestus*

Type	Family	Name	Source
(-)ssRNA	*Orthomyxoviridae*	Culex orthomyxo-like virus	[4, 61]
dsRNA	*Chrysoviridae*	Hubei chryso-like virus 1	[61]
	*Partitiviridae*	Atrato Partiti-like virus 3	[4]
		Beihai partiti-like virus 2	[4, 61]
		Sonnbo virus	[4]
( +)ssRNA	*Picornaviridae*	Ista virus	[4]
	*Virgaviridae*	Alexandroupolis virga-like virus	[61]
dsRNA	*Totiviridae*	Culex inatomii totivirus	[4, 61]
		Fitzroy Crossing toti-like virus	[4]
N/A	Unclassified	Yongsan negev-like virus 1	[4]

Microsporidians of the genus *Cristulospora* have been found in *Cx. modestus* from Uzbekistan [62], and the arthropod parasite *Crithidia brevicula* was found in mosquitoes from the Czech Republic [63]. It has been shown that *Cx. modestus* larvae can be infected with the parasitic mermithid nematode *Romanomermis iyengari* [64] and the fungal parasite *Coelomomyces iliensis* [65]. Further research is needed to characterize the complete microbiome of *Cx. modestus*, including the bacteriome and mycobiome, across a wider geographical landscape.

### Morphology and Identification

#### Morphology

Upon its discovery by Ficalbi in 1889, *Cx. modestus* was described using the phrase “*zanzara di colorito modesto*”, which translates to “*mosquito of modest colouring*”. This is likely the origin of the *modestus* name, as this species is small with homogeneously brown features. The morphology of *Cx. modestus* and other members of the *Barraudius* Edwards subgenus is described in detail by Becker et al. 2010 [10].

**Fig. 2:**
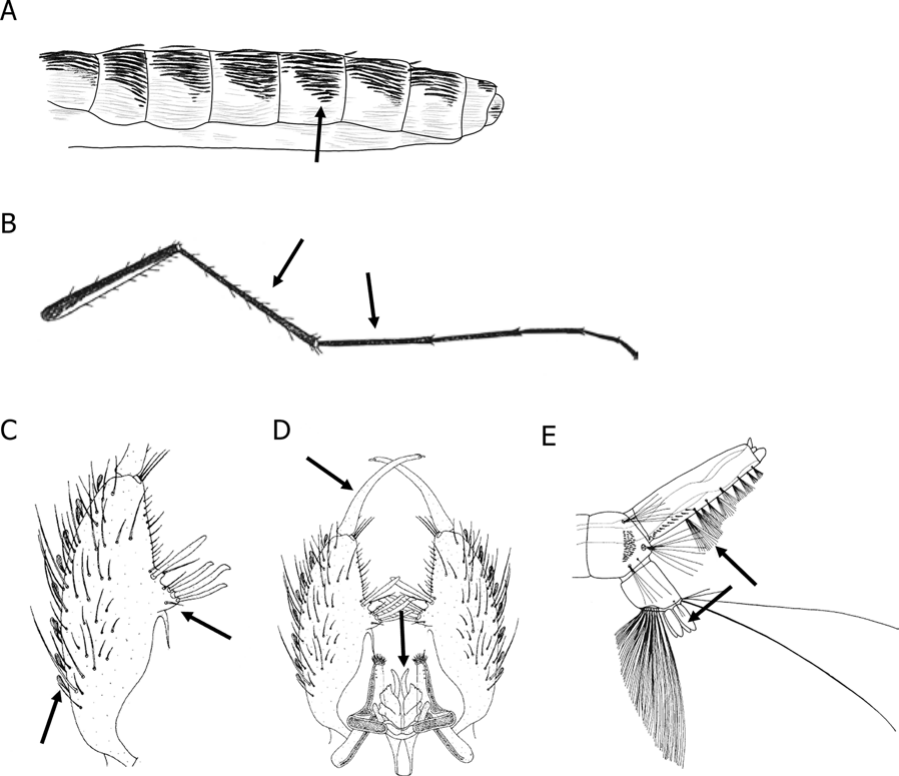
Identifiable characteristics of *Culex modestus*. **A** Schematic of adult abdomen; **B** adult hind leg; **C** male gonocoxite; **D** male hypopygium; **E** larval siphon. Schematic A made using Procreate.com. Schematics **B**–**E** were reproduced and adapted from Becker et al. 2010 [10] with permission from Springer Nature. The black arrows indicate the unique identifiable features

The unique characteristics of *Cx. modestus* that are used for their morphological identification can be found in the terga, hind legs, male gonocoxite, and larval siphons [10]. The terga display dark triangular patches over the longitudinal abdominal bands (Fig. 2A). In the hind legs, tarsomere I is distinctly shorter than the hind tibia (Fig. 2B) [10]. A microscopic image of adult *Cx. modestus* compared to *Cx. pipiens pipiens* can be found in the supplemental material (Additional file 1: Fig. S1). The male gonostylus is long, comprising half the length of the gonocoxite, and the ventral arm of the aedeagus is short, not extending beyond the apex of the paraproct (Fig. 2C–D) [10]. In the larval siphon, the tufts 1-S are arranged in a “ventral zig zag row” towards the apex of the siphon, with one tuft usually inserted in the pecten, and the saddle seta 1-X have two or three branches (Fig. 2E) [10]. Due to general similarities in morphology, *Cx. modestus* can be confused with *Ae. cinereus* or *Cx. martini* [10].

#### Molecular identification

Currently, the primary method of molecular detection of *Cx. modestus* is DNA barcoding. This entails PCR amplification of a target sequence such as the mitochondrial COX1 or ribosomal ITS or rDNA genes to produce a “universal” fragment, which, when sequenced, can be used to identify a range of insect species [66]. A common barcoding target is the 710-bp fragment in COX1 described by Folmer et al. [67]. COX1 is the largest of the mitochondrial subunit-encoding genes and is highly conserved with a slow rate of nucleotide substitution, making it an excellent target for insect species identification. Diagnostic enzyme markers are another target for molecular identification, as malate dehydrogenase 2 and adenylate kinase have been identified as suitable allozyme markers for the taxonomy of *Cx. modestus* [68].

Other *Culex* species have several molecular markers that can be used for identification, including microsatellite loci (e.g. CQ11), nuclear genes such as *ace-2* [69], and the ribosomal ITS region. While morphological identification is considered the gold standard method for mosquito identification, morpho-taxonomy can be highly time-consuming and often misleading if samples are handled improperly or if performed by an inexperienced taxonomist. For large-scale studies capturing high volumes of mosquitoes, a PCR- or RT-PCR-based method of identification would be ideal.

### Disease transmission

Vector incrimination is necessary to determine if an arthropod species is a natural vector of disease. Following the same principle as Koch’s postulates, vector incrimination determines if there is an association in space and time between the arthropod species and human cases of the disease, evidence of direct contact between the arthropod species and the humans, and evidence that the arthropod can harbour and transmit the pathogen [70]. While there is significant evidence of *Cx. modestus* attraction and willingness to bite humans, eco-epidemiological modelling data for this species are lacking. Understanding vector population dynamics and transmission risk over space and time is needed to anticipate potential outbreaks and evaluate the impact of vector control or outbreak mitigation strategies.

The ability of a species to acquire, maintain, and transmit a pathogen is defined as vector competence. Vector competence studies of mosquitoes typically involve measures of infection rate, dissemination rate, and transmission rate as the proportion of mosquitoes with pathogens in the body, head, and saliva, respectively. Mosquitoes with detectable pathogens in the saliva are considered competent vectors, with the proportion of competent mosquitoes in a species expressed as the transmission efficiency. Ideally, mosquito vector competence is determined using living mosquitoes to determine the presence of infectious pathogens in the saliva. Vector competence studies are preferable to the detection of DNA or RNA in field-captured mosquitoes, as the detection of nucleic acid alone is not sufficient evidence to implicate an insect as a vector. However, to understand the true risk of mosquito-borne disease transmission in a given area, measures of vector competence of a species can be used to determine their vectorial capacity. Vectorial capacity, or the Ross-Macdonald model, is defined as the number of infectious mosquito bites per day, a rate comparable to the basic reproduction number (R_0_) [71]. Vectorial capacity includes the human biting rate, the ratio of mosquitoes to humans (or animals), the vector’s daily probability of survival, the extrinsic incubation period of the pathogen, and the vector competence. Currently, there are no estimated measures of *Cx. modestus* vectorial capacity anywhere in the world, but measures of vector competence can serve as a proxy for estimated transmission risk.

*Culex modestus* is a potential vector for several pathogenic viruses and parasites (Table 4). Evidence from epidemiological modelling, vector competence studies, and field detection data support the case that *Cx. modestus* is a competent vector for West Nile virus. Additionally, the evidence that this species is a vector for Usutu virus, avian malaria, and dirofilarial worms is compelling. However, several pathogens have only been detected via RNA or DNA screenings of field-captured *Cx. modestus*, including Japanese encephalitis virus [33], Sindbis virus [72], Kyzylagach virus [73], Tahyna virus [17, 39, 74, 75], Batai virus [76], Lednice virus [77], Ebinur Lake virus (previously named Abbey Lake virus) [78, 79], Zaliv Terpeniya virus [80], Banna virus [74, 81], and *Haemoproteus* sp. [34]. However, whether *Cx. modestus* is a competent vector for these pathogens requires further investigation.

**Table 4:** Reports of vector competence or molecular detection of infectious pathogens in *Culex modestus*

	Family	Pathogen	Detection method	Mosquito type	Country	Source
Virus	*Flaviviridae*	West Nile virus	RNA detection	Field	Czech Republic	[9, 15, 16, 102]
			RNA detection	Field	Italy	[103]
			RNA detection	Field	Kazakhstan	[104]
			RNA detection	Field	Romania	[72]
			RNA detection	Field	Russia	[48, 87]
			Vector competence	Lab	China	[44]
			Vector competence	Lab	France	[42, 43]
		Usutu virus	RNA detection	Field	Czech Republic	[16, 89]
			Vector competence	Field	Belgium	[59]
		Japanese encephalitis virus	RNA detection	Field	China	[33]
	*Alphaviridae*	Sindbis virus	RNA detection	Field	Romania	[72]
		Kyzylagach virus	RNA detection	Field	Czech Republic	[73]
	*Peribunyaviridae*	Tahyna virus	Immunological detection	Field	France	[39]
			Immunological & RNA detection	Field	China	[74]
			Immunological detection	Field	Czech Republic	[17, 75]
		Batai virus	RNA detection	Field	Germany	[76]
		Lednice virus	RNA detection	Field	Czech Republic	[77]
		Ebinur Lake virus (Abbey Lake virus)	RNA detection	Field	China	[78, 79]
	*Phenuiviridae*	Zaliv Terpeniya virus	RNA detection	Field	Azerbaijan	[80]
	*Reoviridae*	Banna virus	RNA detection	Field	China	[74, 81]
Eukaryota	*Onchocercidae*	*Dirofilaria immitis*	Vector competence	Field	Italy	[90]
	*Plasmodiidae*	*Plasmodium relictum*	Vector competence	Lab	Bulgaria	[14]
		*Plasmodium* sp.	DNA detection	Field	Romania	[92]
			DNA detection	Field	Spain	[34]
	*Haemoproteidae*	*Haemoproteus* sp.	DNA detection	Field	Spain	[34]
	*Trypanosomatidae*	*Trypanosoma corvi/culicavium*	Microscopic examination	Field	Czech Republic	[63]

West Nile virus (WNV) is a member of the *Flaviviridae* family and the Japanese encephalitis serocomplex. The virus is maintained in an enzootic transmission cycle between avians and mosquito vectors and can cause severe neuroinvasive disease in humans and other mammals. WNV was first discovered in Uganda in 1937 and is widespread across the globe, causing seasonal outbreaks in temperate regions. The largest recorded outbreak of WNV occurred in the USA in 2002 with more than 4156 human cases, 2942 reports of meningoencephalitis, and 284 deaths [82]. Climatic and environmental shifts driven by the effects of climate change are expected to increase the incidence and spread of WNV in the future [83]. Epidemiological data from Southern France suggested that *Cx. modestus* was the main amplifier of WNV over *Cx. pipiens* s.l. [84], though equine WNV outbreaks in Italy were significantly associated with suitable habitats for both *Cx. pipiens* s.l. and *Cx. modestus* [85]. In contrast to these studies, *Cx. modestus* was estimated to be a less significant amplifier of equine WNV in Croatia than *Cx. pipiens* s.l. and *Ae. vexans* due to their low abundance [86]. The estimated relative risk for WNV transmission was on average 35.2 for *Cx. pipiens* s.l., 65.4 for *Ae. vexans*, and only 2.0 for *Cx. modestus*. In a study that used laboratory-colonized mosquitoes from France, *Cx. modestus* was shown to have a higher vector competence for WNV than *Cx. pipiens pipiens* [43]. The dissemination and transmission rates were 89.2% and 54.5% for *Cx. modestus* and 38.5% and 15.8% for *Cx. pipiens pipiens*, respectively. In contrast, a different study of laboratory colonies reported a lower WNV vector competence in *Cx. modestus* (35% transmission rate) compared to *Cx. pipiens pallens* from Beijing, China (48% transmission rate) [44]. A surveillance study in Russia found a higher WNV infection rate in field-caught *Cx. modestus* than in *Cx. pipiens pipiens* (2.72% vs. 0.79%), despite having a much lower sample size (370 *Cx. modestus* vs. 1261 *Culex pipiens pipiens*) [87]. In Romania, *Cx. modestus* had an infection rate of 1.81 per 1000 mosquitoes for WNV, whereas *Cx. pipiens* s.l. had an infection rate of 0.77 per 1000 mosquitoes [72]. *Culex modestus* from Russia had an infection rate estimate of 0.24 per 1000 mosquitoes, lower than that of *Cx. pipiens* s.l. (0.51 per 1000 mosquitoes) [48]. These data combined suggest *Cx. modestus* may play an important role in WNV circulation.

Usutu virus (USUV) is another flavivirus antigenically similar to WNV. USUV rarely causes symptomatic infections in humans but can result in rapid and destructive outbreaks among birds [88]. RNA of Usutu virus has been detected in pools of *Cx. modestus* captured from the Czech Republic [16, 89]. Another study using field-captured mosquitoes from Belgium investigated the vector competence of *Cx. modestus* to USUV [59]. The sample size of *Cx. modestus* included in the latter study was low (*n *= 5) but the transmission potential for USUV was high (20% transmission efficiency), suggesting that *Cx. modestus* could be potent vectors for USUV. The co-circulation of WNV and USUV in the same habitat has been reported in the Czech Republic [16], but given that these two arboviruses share considerable geographical overlap, co-circulation is likely not an uncommon occurrence. Because of their preferred habitat for rural wetland areas, farmers, hunters, and people living around ponds and farmland animals, such as horses, may have a higher risk of WNV or USUV infection by *Cx. modestus* [9].

*Culex modestus* has been implicated as a vector of the parasitic heartworm *Dirofilaria immitis* with a vector efficiency index of 2.0, higher than that of *An. maculipennis* (1.4) and *Cx. pipiens* s.l. (1.2), but lower than in *Ae. caspius* (6.3) [90]. Data suggest that these four species including *Cx. modestus* were primarily responsible for the transmission of canine filariasis in Piedmont, Italy [91]. Field *Cx. modestus* captured from Spain were found positive for avian malaria (*Plasmodium* sp., 0.5–6.3% prevalence) and *Haemoproteus* sp. (unknown prevalence) [34]. In mosquitoes captured from Romania, one positive pool of *Cx. modestus* containing *Plasmodium* sp. was found [92]. In addition, there is evidence from a laboratory investigation that a small number of *Cx. modestus* could transmit *Plasmodium relictum* sporozoites in the salivary glands [14]. In a survey of blood-sucking insects in the Czech Republic, *Cx. modestus* were found carrying the avian trypanosome *Trypanosoma corvi/culicavium* [63].

### Capture and control

#### Trapping methods

Wild *Cx. modestus* have been captured using a variety of different trapping methods, including HLCs [32, 51, 52], BG-Sentinel traps baited with CO_2_ [4, 13, 59, 93], CDC light traps baited with CO_2_ [12, 15, 26, 52, 53, 61], Mosquito Magnet Pro traps [19, 28, 29], and larval dipping [18, 19, 21, 29, 94]. Other trapping methods include CO_2_-baited traps (undescribed) [32, 95], CO_2_-baited CDC traps without light [35], CDC traps without light baited with a Japanese quail or rabbit [9], CO_2_-baited CDC traps with sentinel birds (Japanese quail or chicken) or sentinel mammals (rabbit or guinea pig) [35], the EVS/CO_2_ Mosquito Trap [14], and a resting box baited with Red-footed falcon nestlings [96].

In studies that have compared trapping methods, either a majority or an equal number of *Cx. modestus* adults could be captured with HLCs or CO_2_-baited traps. The only exception is a study performed in the UK which captured significantly higher proportions of *Cx. modestus* with the Mosquito Magnet Pro than with resting boxes [28]. Regarding *Cx. pipiens* s.l., more *Cx. modestus* were captured with HLCs than with CO_2_-baited traps in Italy [32]. In France, more *Cx. modestus* were captured with CO_2_-baited traps (38 mosquitoes per trap night) and HLCs (33 mosquitoes per trap night) than with pigeon-baited traps (0.3 mosquitoes per trap night), and no *Cx. modestus* were caught using resting boxes (336 trap nights) or gravid traps (176 trap nights) [95]. Similar to *Cx. modestus*, more *Cx. pipiens* s.l. were captured using CO_2_-baited traps than the pigeon-baited traps (78 vs. 7 mosquitoes per trap night), despite this species being highly ornithophilic, suggesting that CO_2_-baited traps are more attractive to these species than the use of live birds. Overall, the CO_2_-baited traps collected two-thirds of all mosquitoes as well as sampled all the 14 species identified in the study [95]. A much lower number of *Cx. pipiens* s.l. were caught with HLCs than *Cx. modestus* (3 vs. 33 mosquitoes per trap night), suggesting that *Cx. modestus* is more attracted to humans than *Cx. pipiens* s.l. [95]. Similarly, a study from China captured an equal number of *Cx. modestus* in CO_2_-baited CDC light traps (*n* = 260) and HLCs (*n* = 258) [52]. Interestingly, although *Cx. modestus* could be captured using human bait, no *Cx. pipiens molestus* were caught using this technique, despite the anthropophilic behaviour of the *molestus* biotype, whereas they could be caught with CO_2_-baited CDC light traps (*n* = 267) [52].

Traps can be coupled with lures or attractants to enhance mosquito attraction. CO_2_, released by dry ice or fermentation, simulates animal respiration, which is a long-distance attractant for mosquitoes. BG lure and BG Sweetscent (Biogents AG, Regensburg, Germany) mimic the scent of human and/or animal skin and can be coupled with CO_2_-baited and/or light traps. The Mosquito Magnet Pro trap uses Octenol as a lure, which selectively attracts mammalophilic species*. Culex modestus* was the dominant mosquito species captured using the Mosquito Magnet Pro in a surveillance study in the UK compared to *Cx. pipiens* s.l.*/Cx. torrentium*, suggesting that *Cx. modestus* is more attracted to mammalian hosts than the latter species [29]. In a different study, CDC light traps (without light) baited with CO_2_ from dry ice captured 97% of all *Cx. modestus* mosquitoes compared to horse urine, acetone, O-1-octen-3-ol, and ammonium hydroxide [93].

Depending on the purpose of the research, when aiming to capture high densities of *Cx. modestus*, CO_2_-baited traps could be the best choice as they are less labour-intensive than HLCs. When the aim is to capture host-seeking adults that are specifically human-biting, HLC is the most suitable method; otherwise, the use of a trap coupled with a human- or animal-scented lure would be appropriate.

#### Insecticides and insecticide resistance

In the event of an arbovirus outbreak, interventions should be carefully chosen to target mosquito populations responsible for transmission. Insecticides delivered through, for example, space spraying can be used to kill adult vectors, while other interventions such as larval source management can be used to reduce the density of vector populations. Currently, insecticide research specifically targeting *Cx. modestus* is scarce. Exposure to 5 mg/l avermectin-impregnated fine plant powder was significantly toxic to *Cx. modestus* larvae [97]. Two water-based pyrethroid formulations (Aqua-K-Othrine, 2% deltamethrin, and Pesguard S102, 10% d-phenothrin) were tested for their potential use in aerial spraying to control adult mosquitoes, of which both pyrethroids showed efficacy against *Cx. modestus* and other riceland mosquitoes in Greece [45]. Space spraying campaigns to target adult host-seeking mosquitoes would be best carried out during the peak hours of the night when the mosquitoes are most active [52].

The widespread use of insecticides for the control of agricultural pests and disease vectors has led to a global rise in genetic and phenotypic insecticide resistance in mosquitoes. Only one study from Belgium has reported the presence of insecticide resistance markers in *Cx. modestus* [98]. In 51 captured mosquitoes, knockdown resistance to pyrethroids and DDT (L1014F) was 43%, while the acetylcholinesterase-1 substitution conferring resistance to organophosphates and carbamates (G119S) was 3.9% [98]. Insecticide-resistant mosquito populations may be less susceptible to vector control responses that are insecticide-based, and there is also evidence that resistance mutations can increase arbovirus dissemination in the mosquito [99]. It is important to monitor insecticide resistance in areas with known or suspected mosquito-borne pathogen transmission.

## Conclusion

Despite their extensive presence and capacity to spread human pathogens, little is known about *Cx. modestus*. These mosquitoes are widespread across the European continent and parts of Asia and northern Africa, but their origin and migration patterns are insufficiently described. *Culex modestus* are adapted to temperate regions, thriving during the warm summer months whilst able to survive cold winters. This species feeds on a wide range of bird species, but nonetheless appears to be highly attracted to humans, suggesting it could be a model ‘bridge’ vector for enzootic pathogens. While this species is likely an intermediate vector for West Nile virus, Usutu virus, canine filariasis, and avian malaria, more research is needed to implicate *Cx. modestus* in the transmission of other viruses and parasites. For example, Sindbis virus, Tahyna virus, and *Trypanosoma* sp. have been detected in field-captured *Cx. modestus*, but no studies on their vector competence for these pathogens have been carried out. *Culex modestus* are known to harbour insect-specific viruses, but limited knowledge is available on the bacteria or fungi that colonize them. It would be especially interesting to investigate whether *Wolbachia* has any effect on the life history traits or vector competence for this species. While *Cx. modestus* appears to be a rural pest, it remains unclear whether these mosquitoes prefer feeding indoors or outdoors and how well they survive in more urban areas. Mosquitoes captured from Belgium were found to be insecticide-resistant to all four major classes of insecticides used in public health, while it is unknown if mosquitoes from other areas carry these adaptations, potentially complicating future vector control strategies. Although *Cx. modestus* is a potential vector for human pathogens, there is still a significant lack of baseline knowledge on this species. More research on the biology and behaviour of *Cx. modestus* is thus urgently needed. As a priority, the vectorial capacity of *Cx. modestus* to WNV in hotspot areas should be determined (including measures of mosquito abundance, human-biting rate, etc.) to implicate this species as a capable WNV vector. Mosquito abundance studies can be done in parallel to research into the behaviour of *Cx. modestus*, for example to determine feeding patterns (endophagy vs. exophagy), resting preferences (endophily vs. exophily), and breeding sites (urban vs. rural), which can be used to determine optimal target sites for mosquito control. Finally, if this species is confirmed as an efficient vector of WNV, cost-effective methods of reducing populations of *Cx. modestus*, such as larval source reduction or insecticide-based interventions, should be investigated for WNV outbreak prevention and mitigation.

### Supplementary Information

**Additional file 1: Fig. S1.** Comparison of **A** *Culex pipiens pipiens* and **B** *Culex* *modestus*. The black arrows indicate the unique characteristics for morphological identification of Culex modestus.

**Additional file 2: Table S1**. Studies with reports of *Culex modestus* by country.

## Data Availability

All data generated or analyzed during this study are included in this published article and its supplementary information files.

## Abbreviations

*Ae.*: *Aedes*
*An.*: *Anopheles*
CDC: Centers for Disease Prevention and Control
COX1: Cytochrome oxidase 1
*Cs*.: *Culiseta*
*Cx*.: *Culex*
ECDC: European Centre for Disease Prevention and Control
HLC: Human landing catch
ISV: Insect-specific virus
RBRI: Relative biting risk index
RH: Relative humidity
S.l.: Sensu lato
*Ur*.: *Uranotaenia*
USUV: Usutu virus
WNV: West Nile virus
