# Supplementary figures and images for: Culex modestus: the overlooked mosquito vector
Source: Parasit Vectors. 2023 Oct 20;16:373. doi: 10.1186/s13071-023-05997-6 (PMC10588236; doi:10.1186/s13071-023-05997-6)

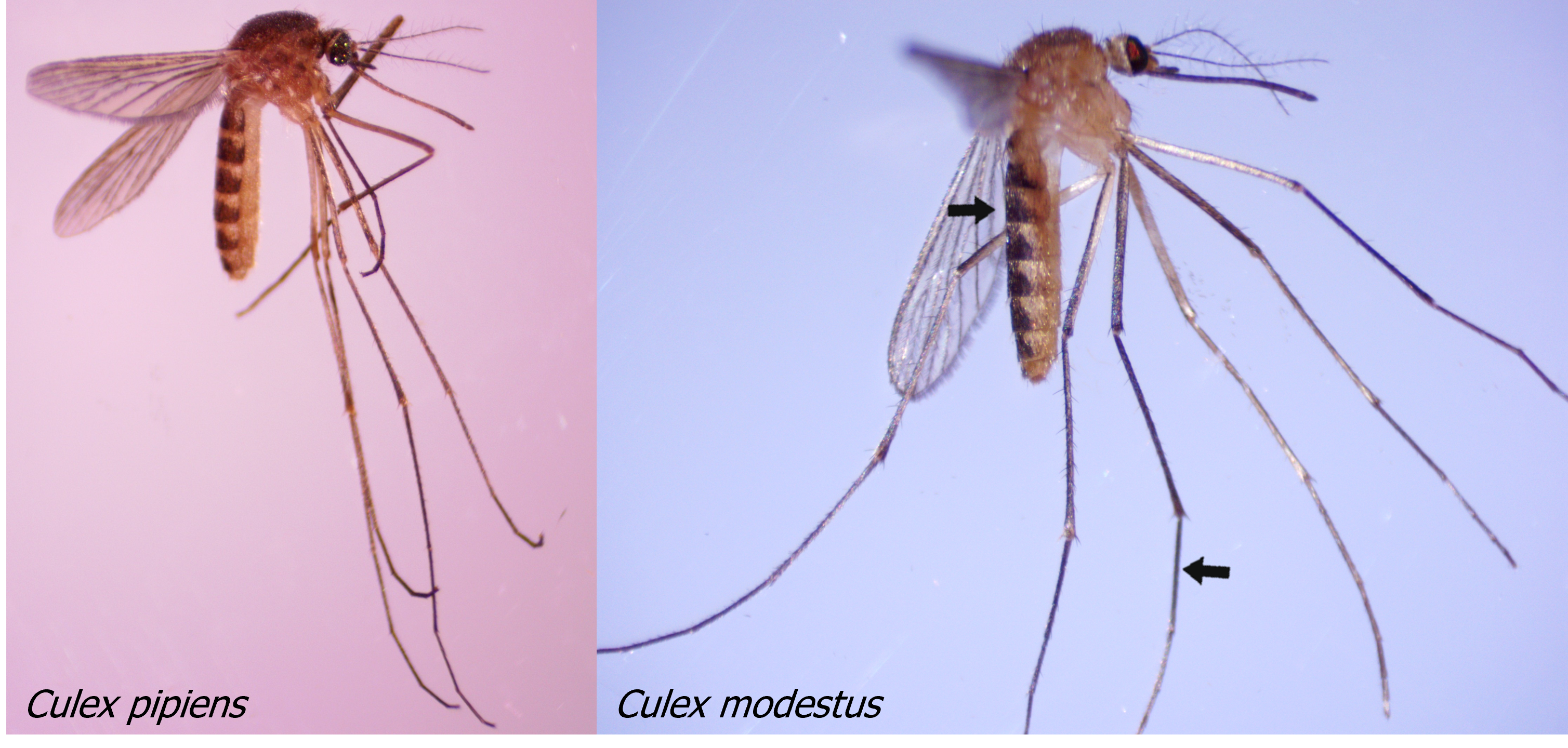

Supplement: Supplementary file 1 — Additional file 1: Fig. S1. Comparison of A Culex pipiens pipiens and B Culex modestus. The black arrows indicate the unique characteristics for morphological identification of Culex modestus. [file 13071_2023_5997_MOESM1_ESM.tif]
