# Supplementary material for: Culex modestus: the overlooked mosquito vector
Source: Parasit Vectors. 2023 Oct 20;16:373. doi: 10.1186/s13071-023-05997-6 (PMC10588236; doi:10.1186/s13071-023-05997-6)
Supplement: Supplementary file 2 — Additional file 2: Table S1. Studies with reports of Culex modestus by country. [file 13071_2023_5997_MOESM2_ESM.docx]

**Table S1** Studies with reports of *Culex modestus* by country.

| **Continent** | **Country** | **Reference** |
| --- | --- | --- |
| Europe | Albania | [1] |
|  | Austria | [1,2] |
|  | Belarus | [1] |
|  | Belgium | [3–6] |
|  | Bulgaria | [7] |
|  | Croatia | [8,9] |
|  | Czech Republic | [10–22] |
|  | Denmark | [23] |
|  | Finland | [24] |
|  | France | [25–32] |
|  | Georgia | [1] |
|  | Germany | [33,34] |
|  | Greece | [35–38] |
|  | Hungary | [39] |
|  | Italy | [40–52] |
|  | Macedonia | [1] |
|  | Moldova | [53,54] |
|  | Montenegro | [1] |
|  | Netherlands | [1,2] |
|  | Poland | [55] |
|  | Portugal | [56] |
|  | Romania | [57–60] |
|  | Serbia | [1,2] |
|  | Slovakia | [1,2] |
|  | Slovenia | [1] |
|  | Spain | [61–68] |
|  | Sweden | [69] |
|  | Switzerland | [1,2] |
|  | Ukraine | [1,2] |
|  | United Kingdom | [70–76] |
| Europe/Asia | Azerbaijan | [77] |
|  | Kazakhstan | [78] |
|  | Russia | [79–83] |
|  | Turkey | [84] |
| Asia | Afghanistan | [85] |
|  | China | [86–94] |
|  | India | [95] |
|  | Iran | [96–103] |
|  | Iraq | [85,104] |
|  | Israel | [1] |
|  | Mongolia | [105] |
|  | Pakistan | [105] |
|  | Tajikistan | [105] |
|  | Uzbekistan | [106,107] |
| Africa | Algeria | [108] |
|  | Morocco | [1,2] |

References

[1] Robert V, Gunay F, Le Goff G, et al. Distribution chart for Euro-Mediterranean mosquitoes (western Palaearctic region). Journal of the European Mosquito Control Association 2019;37:1–28.

[2] European Centre for Disease Prevention and Control (ECDC). Mosquito maps 2023. https://www.ecdc.europa.eu/en/disease-vectors/surveillance-and-disease-data/mosquito-maps (accessed July 26, 2023).

[3] De Wolf K, Vanderheyden A, Deblauwe I, et al. First record of the West Nile virus bridge vector *Culex modestus* Ficalbi (Diptera: Culicidae) in Belgium, validated by DNA barcoding. Zootaxa 2021;4920:131–9.

[4] Wang L, Rosales Rosas AL, De Coninck L, et al. Establishment of *Culex modestus* in Belgium and a Glance into the Virome of Belgian Mosquito Species. MSphere 2021;6.

[5] Wang L, Soto A, Remue L, et al. First Report of Mutations Associated With Pyrethroid (L1014F) and Organophosphate (G119S) Resistance in Belgian *Culex* (Diptera: Culicidae) Mosquitoes. Journal of Medical Entomology 2022;59:2072–9.

[6] Soto A, De Coninck L, Devlies AS, et al. Belgian *Culex pipiens pipiens* are competent vectors for West Nile virus while *Culex modestus* are competent vectors for Usutu virus. PLOS Neglected Tropical Diseases 2023;17:e0011649.

[7] Dimitrov D, Bobeva A, Marinov MP, et al. First evidence for development of *Plasmodium relictum* (Grassi and Feletti, 1891) sporozoites in the salivary glands of *Culex modestus* Ficalbi, 1889. Parasitology Research 2023:1–5.

[8] Merdić E, Jeličić Ž, Krčmar S, et al. Efficacy of mosquito attractants in various habitats of a floodplain. Biologia 2010;65:545–51.

[9] Vignjević G, Vrućina I, Sestak I, et al. Equine seroprevalence rates as an additional indicator for a more accurate risk assessment of the West Nile virus transmission. Coll Antropol 2013;37:949–56.

[10] Danielová V, Hájková Z, Kolman JM, et al. [Results of the virological examination of mosquitoes in southern Moravia in 1962-1964]. Cesk Epidemiol Mikrobiol Imunol 1966;15:178–84.

[11] Danielová V & Holubová J. Two more mosquito species proved as vectors of Tahyna virus in Czechoslovakia. Folia Parasitol (Praha) 1977;24:187–9.

[12] Hönig V, Palus M, Kaspar T, et al. Multiple lineages of Usutu virus (*Flaviviridae, Flavivirus*) in blackbirds (*Turdus merula*) and mosquitoes (*Culex pipiens, Cx. modestus*) in the Czech Republic (2016–2019). Microorganisms 2019;7.

[13] Hubálek Z, Rudolf I, Bakonyi T, et al. Mosquito (Diptera: Culicidae) surveillance for arboviruses in an area endemic for West Nile (Lineage Rabensburg) and Tahyna viruses in Central Europe. Journal of Medical Entomology 2010;47:466–72.

[14] Radrova J, Seblova V, & Votypka J. Feeding Behavior and Spatial Distribution of *Culex* Mosquitoes (Diptera: Culicidae) in Wetland Areas of the Czech Republic. Journal of Medical Entomology 2013;50:1097–104.

[15] Rudolf I, Bakonyi T, Šebesta O, et al. West Nile virus lineage 2 isolated from *Culex modestus* mosquitoes in the Czech Republic, 2013: expansion of the European WNV endemic area to the North? Eurosurveillance 2014;19:20867.

[16] Rudolf I, Bakonyi T, Šebesta O, et al. Co-circulation of Usutu virus and West Nile virus in a reed bed ecosystem. Parasites and Vectors 2015;8:1–5.

[17] Rudolf I, Blažejová H, Šebesta O, et al. West Nile virus (lineage 2) in mosquitoes in southern Moravia - awaiting the first autochthonous human cases. Epidemiol Mikrobiol Imunol 2018;67:44–6.

[18] Rudolf I, Šikutová S, Šebesta O, et al. Overwintering of *Culex modestus* and other mosquito species in a reedbed ecosystem, including arbovirus findings. Journal of the American Mosquito Control Association 2020;36:257–60.

[19] Šebesta O, Gelbič I, & Peško J. Seasonal dynamics of mosquito occurrence in the Lower Dyje River Basin at the Czech-Slovak-Austrian border. Italian Journal of Zoology 2013;80:125–38.

[20] Šikutová S, Dočkal P, Straková P, et al. First record of mosquito-borne kyzylagach virus in central Europe. Viruses 2020;12.

[21] Svobodova M, Volf P, & Votypka J. Trypanosomatids in ornithophilic bloodsucking Diptera. Medical and Veterinary Entomology 2015;29:444–7.

[22] Votýpka J, Seblová V, & Rádrová J. Spread of the West Nile virus vector *Culex modestus* and the potential malaria vector *Anopheles hyrcanus* in central Europe. J Vector Ecol 2008;33:269–77.

[23] Bødker R, Klitgård K, Byriel DB, et al. Establishment of the West Nile virus vector, *Culex modestus* , in a residential area in Denmark. Journal of Vector Ecology 2014;39:1–3.

[24] Culverwell C l. & Vapalahti O. First record of *Culex modestus* in Finland. Journal of the European Mosquito Control Association 2023:1–4.

[25] Balenghien T, Fouque F, Sabatier P, et al. Horse-, Bird-, and Human-Seeking Behavior and Seasonal Abundance of Mosquitoes in a West Nile Virus Focus of Southern France. Journal of Medical Entomology 2006;43:936–46.

[26] Balenghien T, Vazeille M, Reiter P, et al. Evidence of laboratory vector competence of *Culex modestus* for West Nile virus. Journal of the American Mosquito Control Association 2007;23:233–6.

[27] Cailly P, Balenghien T, Ezanno P, et al. Role of the repartition of wetland breeding sites on the spatial distribution of *Anopheles* and *Culex*, human disease vectors in Southern France. Parasites and Vectors 2011;4:65.

[28] Callot J & Van-Ty D. Localités françaises nouvelles pour *Culex* (*Culex*) *theileri*), *Culex* (*Barraudius*) *modestus* et *Culex* (*Neoculex*) *impudicus*. Ann Parasitol Hum Comp 1942;19:142–50.

[29] Chippaux A, Rageau J, & Mouchet J. [Hibernation of arbovirus Tahyna in Culex modestus Fic. in France]. C R Acad Hebd Seances Acad Sci D 1970;270:1648–50.

[30] L’Ambert G, Ferré J-B, Schaffner F, et al. Comparison of different trapping methods for surveillance of mosquito vectors of West Nile virus in Rhône Delta, France. Journal of Vector Ecology 2012;37:269–75.

[31] Ponçon N, Toty C, L'Ambert G, et al. Population dynamics of pest mosquitoes and potential malaria and West Nile virus vectors in relation to climatic factors and human activities in the Camargue, France. Med Vet Entomol. 2007 Dec 18;21(4):350–7.

[32] Pradel JA, Martin T, Rey D, et al. Is *Culex modestus* (Diptera: Culicidae), Vector of West Nile Virus, Spreading in the Dombes Area, France? Journal of Medical Entomology 2009;46:1269–81.

[33] Krüger A, Börstler J, Badusche M, et al. Mosquitoes (Diptera: Culicidae) of metropolitan Hamburg, Germany. Parasitology Research 2014;113:2907–14.

[34] Scheuch D, Schäfer M, Eiden M, et al. Detection of Usutu, Sindbis, and Batai Viruses in Mosquitoes (Diptera: Culicidae) Collected in Germany, 2011–2016. Viruses 2018;10:389.

[35] Chaskopoulou A, Latham MD, Pereira RM, et al. Efficacy of aerial ultra-low volume applications of two novel water-based formulations of unsynergized pyrethroids against riceland mosquitoes in Greece. Journal of the American Mosquito Control Association 2011;27:414–22.

[36] Chaskopoulou A, Dovas CI, Chaintoutis SC, et al. Detection and early warning of West Nile virus circulation in Central Macedonia, Greece, using sentinel chickens and mosquitoes. Vector-Borne and Zoonotic Diseases 2013;13:723–32.

[37] Konstantinidis K, Dovrolis N, Kouvela A, et al. Defining virus-carrier networks that shape the composition of the mosquito core virome of a local ecosystem. Virus Evolution 2022;8:veac036.

[38] Köseoğlu AE, Paltacı S, Can H, et al. Applicability evaluation of mtDNA based molecular identification in mosquito species/subspecies/biotypes collected from Thessaloniki, Greece. Veterinary Parasitology: Regional Studies and Reports 2023;41:100869.

[39] Soltész Z, Erdélyi K, Bakonyi T, et al. West Nile virus host-vector-pathogen interactions in a colonial raptor. Parasites and Vectors 2017;10.

[40] Ascoli V, Facchinelli L, Valerio L, et al. Distribution of mosquito species in areas with high and low incidence of classic Kaposi’s sarcoma and seroprevalence for HHV-8. Medical and Veterinary Entomology 2006;20:198–208.

[41] Bellini R, Veronesi R, Draghetti S, et al. Study on the flying height of *Aedes caspius* and *Culex pipiens* females in the Po Delta area, Italy. J Am Mosq Control Assoc 1997;13:356–60.

[42] Bisanzio D, Giacobini M, Bertolotti L, et al. Spatio-temporal patterns of distribution of West Nile virus vectors in eastern Piedmont Region, Italy. Parasites and Vectors 2011;4:230.

[43] Ficalbi E. Notizie preventive sulle zanzare italiane. IV. Nota preventiva. Descrizione di una specie nuova. Zanzara di colorito modesto *Culex modestus*, n. sp. Bullettino Della Società Entomologica Italiana 1890;21:93–4.

[44] Latrofa MS, Montarsi F, Ciocchetta S, et al. Molecular xenomonitoring of *Dirofilaria immitis* and *Dirofilaria repens* in mosquitoes from north-eastern Italy by real-time PCR coupled with melting curve analysis. Parasites and Vectors 2012;5:76.

[45] Mancini G, Montarsi F, Calzolari M, et al. Mosquito species involved in the circulation of West Nile and Usutu viruses in Italy. Veterinaria Italiana 2017;53:97–110.

[46] Monaco F, Goffredo M, Briguglio P, et al. Descrizione dei focolai di west nile disease nel 2011 nella regione Sardegna, Italia. Veterinaria Italiana 2015;51:5–16.

[47] Montarsi F, Mazzon L, Cazzin S, et al. Seasonal and Daily Activity Patterns of Mosquito (Diptera: Culicidae) Vectors of Pathogens in Northeastern Italy. Journal of Medical Entomology 2015;52:56–62.

[48] Pollono F, Rossi L, & Cancrini G. [Research on Culicidae attracted to dog bait in Piedmont]. Parassitologia 1998;40:439–45.

[49] Ricci I, Cancrini G, Gabrielli S, et al. Searching for *Wolbachia* (Rickettsiales: Rickettsiaceae) in Mosquitoes (Diptera: Culicidae): Large Polymerase Chain Reaction Survey and New Identifications. Journal of Medical Entomology 2002;39:562–7.

[50] Rossi L, Pollono F, Meneguz P, et al. [Four species of mosquito as possible vectors for *Dirofilaria immitis* piedmont rice-fields]. Parassitologia 1999;41:537–42.

[51] Toma L, Cipriani M, Goffredo M, et al. First report on entomological field activities for the surveillance of West Nile disease in Italy. Vet Ital 2008;44:499–512.

[52] Veronesi R, Gentile G, Carrieri M, et al. Seasonal pattern of daily activity of *Aedes caspius*, *Aedes detritus*, *Culex modestus*, and *Culex pipiens* in the Po Delta of northern Italy and significance for vector-borne disease risk assessment. Journal of Vector Ecology 2012;37:49–61.

[53] Sulesco TM, Toderas LG, Uspenskaia IG, et al. Larval Habitats Diversity and Distribution of the Mosquito (Diptera: Culicidae) Species in the Republic of Moldova. Journal of Medical Entomology 2015;52:1299–308.

[54] Sulesco T, Toderas L, & Toderas I. A recent survey of the mosquito (Diptera: Culicidae) fauna and seasonal human biting activity in the city of Chisinau, Moldova. Journal of the European Mosquito Control Association 2013.

[55] Wegner E. Mosquito fauna (Diptera: Culicidae) of five different towns in Poland with special reference to the occurence of human disease vectors. Fragmenta Faunistica 2008;51:15–22.

[56] Freitas FB, Novo MT, Esteves A, et al. Species composition and WNV screening of mosquitoes from lagoons in a wetland area of the Algarve, Portugal. Frontiers in Physiology 2012;2.

[57] Ionicǎ AM, Zittra C, Wimmer V, et al. Mosquitoes in the Danube Delta: Searching for vectors of filarioid helminths and avian malaria. Parasites and Vectors 2017;10.

[58] Cotar AI, Falcuta E, Prioteasa LF, et al. Transmission Dynamics of the West Nile Virus in Mosquito Vector Populations under the Influence of Weather Factors in the Danube Delta, Romania. EcoHealth 2016;13:796–807.

[59] Crivei LA, Moutailler S, Gonzalez G, et al. Detection of West Nile Virus Lineage 2 in Eastern Romania and First Identification of Sindbis Virus RNA in Mosquitoes Analyzed using High-Throughput Microfluidic Real-Time PCR. Viruses 2023;15.

[60] Török E, Tomazatos A, Cadar D, et al. Pilot longitudinal mosquito surveillance study in the Danube Delta Biosphere Reserve and the first reports of *Anopheles algeriensis* Theobald, 1903 and *Aedes hungaricus* Mihályi, 1955 for Romania. Parasites and Vectors 2016;9.

[61] Alcaide M, Rico C, Ruiz S, et al. Disentangling vector-borne transmission networks: A universal DNA barcoding method to identify vertebrate hosts from arthropod bloodmeals. PLoS ONE 2009;4.

[62] Bravo-Barriga D, Gomes B, Almeida APG, et al. The mosquito fauna of the western region of Spain with emphasis on ecological factors and the characterization of *Culex pipiens* forms. Journal of Vector Ecology 2017;42:136–47.

[63] Ferraguti M, Martinez-de la Puente J, Muñoz J, et al. Avian *Plasmodium* in *Culex* and *Ochlerotatus* Mosquitoes from Southern Spain: Effects of Season and Host-Feeding Source on Parasite Dynamics. PLOS ONE 2013;8:e66237.

[64] Ferraguti M, Heesterbeek H, Martínez‐de la Puente J, et al. The role of different *Culex* mosquito species in the transmission of West Nile virus and avian malaria parasites in Mediterranean areas. Transboundary and Emerging Diseases 2021;68:920–30.

[65] Martínez-de la Puente J, Ruiz S, Soriguer R, et al. Effect of blood meal digestion and DNA extraction protocol on the success of blood meal source determination in the malaria vector *Anopheles atroparvus*. Malaria Journal 2013;12:109.

[66] Muñoz J, Ruiz S, Soriguer R, et al. Feeding patterns of potential West Nile virus vectors in South-West Spain. PLoS ONE 2012;7.

[67] Roiz D, Eritja R, Escosa R, et al. A survey of mosquitoes breeding in used tires in Spain for the detection of imported potential vector species. Journal of Vector Ecology 2007;32:10.

[68] Roiz D, Ruiz S, Soriguer R, et al. Landscape effects on the presence, abundance and diversity of mosquitoes in mediterranean wetlands. PLoS ONE 2015;10.

[69] Bergman A & Hesson JC. *Wolbachia* prevalence in the vector species *Culex pipiens* and *Culex torrentium* in a Sindbis virus-endemic region of Sweden. Parasites and Vectors 2021;14:428.

[70] Brugman VA, England ME, Stoner J, et al. How often do mosquitoes bite humans in southern England? A standardised summer trial at four sites reveals spatial, temporal and site-related variation in biting rates. Parasites and Vectors 2017;10:420.

[71] Brugman VA, Hernández-Triana LM, England ME, et al. Blood-feeding patterns of native mosquitoes and insights into their potential role as pathogen vectors in the Thames estuary region of the United Kingdom. Parasites and Vectors 2017;10:1–12.

[72] Golding N, Nunn MA, Medlock JM, et al. West Nile virus vector *Culex modestus* established in southern England. Parasites and Vectors 2012;5:32.

[73] Hernández-Triana L, Brugman V, Pramual P, et al. Genetic diversity and population structure of *Culex modestus* across Europe: does recent appearance in the United Kingdom reveal a tendency for geographical spread? Medical and Veterinary Entomology 2019;34.

[74] Marshall JF. Records of *Culex* (*Barraudius*) *modestus* Ficalbi (Diptera, Culicidæ) obtained in the south of England. Nature 1945;156:172–3.

[75] Medlock JM & Vaux AGC. Distribution of West Nile virus vector, *Culex modestus*, in England. Veterinary Record 2012;171:278–278.

[76] Vaux AGC, Gibson G, Hernandez-Triana LM, et al. Enhanced West Nile virus surveillance in the North Kent marshes, UK. Parasites and Vectors 2015;8.

[77] L’vov DK, Al’khovskiĭ SV, Shchelkanov MI, et al. [Genetic characterization of the Zaliv Terpeniya virus (ZTV, Bunyaviridae, Phlebovirus, Uukuniemi serogroup) strains isolated from the ticks *Ixodes* (*Ceratixodes*) *uriae* White, 1852, obligate parasites of the Alcidae birds, in high latitudes of Northern Eurasia and the mosquitoes *Culex modestus* Ficalbi, 1889, in subtropics Transcaucasus]. Vopr Virusol 2014;59:12–8.

[78] Nurmakhanov T, Sansyzbaev Y, Atshabar B, et al. Phylogenetic Characteristics of West Nile Virus Isolated From *Culex modestus* Mosquitoes in West Kazakhstan. Frontiers in Public Health 2021;8.

[79] Belevich O, Yurchenko Y, Alekseev A, et al. Toxic Effects of Fine Plant Powder Impregnated With Avermectins on Mosquito Larvae and Nontarget Aquatic Invertebrates. Journal of Medical Entomology 2021;58:773–80.

[80] Dzerzhinskiĭ VA, Dubitskiĭ AM, & Deshevykh ND. [Appearance of variants in the artificial infection of larvae of the blood-sucking mosquito, *Culex modestus*, infested with the entomopathogenic fungus, *Coelomomyces iliensis*]. Parazitologiia 1975;9:540–2.

[81] Fyodorova MV, Savage HM, Lopatina JV, et al. Evaluation of Potential West Nile Virus Vectors in Volgograd Region, Russia, 2003 (Diptera: Culicidae): Species Composition, Bloodmeal Host Utilization, and Virus Infection Rates of Mosquitoes. Journal of Medical Entomology 2006;43:552–63.

[82] Shaikevich E, Bogacheva A, & Ganushkina L. *Dirofilaria* and *Wolbachia* in mosquitoes (Diptera: Culicidae) in central European Russia and on the Black Sea coast. Parasite 2019;26.

[83] Shaikevich EV, Zagoskin MV, & Mukha DV. Comparative characteristics of the intergenic spacer of the ribosomal RNA gene cluster in mosquitoes of the genus Culex (Diptera: Culicidae). Mol Biol 2013;47:364–72.

[84] Gunay F, Alten B, Simsek F, et al. Barcoding Turkish *Culex* mosquitoes to facilitate arbovirus vector incrimination studies reveals hidden diversity and new potential vectors. Acta Tropica 2015;143:112–20.

[85] Rueda LM, Pecor JE, Lowen RG, et al. New record and updated checklists of the mosquitoes of Afghanistan and Iraq. Jvec 2008;33:397–402.

[86] Cao YX, Fu SH, Tian ZF, et al. [Isolation and identification of Banna virus from mosquito for the first time in Inner Mongolia]. Zhonghua Shi Yan He Lin Chuang Bing Du Xue Za Zhi. 2009 Apr;23(2):106-8.

[87] Cao Y, Fu S, Tian Z, et al. Distribution of mosquitoes and mosquito-borne arboviruses in Inner Mongolia, China. Vector-Borne and Zoonotic Diseases 2011;11:1577–81.

[88] Cao Y, Fu S, Song S, Cai L, Zhang H, Gao L, et al. Isolation and Genome Phylogenetic Analysis of Arthropod-Borne Viruses, Including Akabane Virus, from Mosquitoes Collected in Hunan Province, China. Vector-Borne and Zoonotic Diseases 2019;19:62–72.

[87] Cao Y, Fu S, Tian Z, et al. Distribution of mosquitoes and mosquito-borne arboviruses in Inner Mongolia, China. Vector-Borne and Zoonotic Diseases 2011;11:1577–81.

[90] Jiang SF, Zhang YM, Guo XX, et al. Experimental Studies on Comparison of the Potential Vector Competence of Four Species of *Culex* Mosquitoes in China to Transmit West Nile Virus. Journal of Medical Entomology 2010;47:788–90.

[91] Liu R, Zhang G, Sun X, et al. [Isolation and molecular characterization on Abbey Lake Orthobunyavirus (Bunyaviridae) in Xinjiang, China]. Zhonghua Liu Xing Bing Xue Za Zhi = Zhonghua Liuxingbingxue Zazhi 2014;35:939–42.

[92] Wang ZM, Xing D, Wu ZM, et al. Biting activity and host attractancy of mosquitoes (Diptera: Culicidae) in Manzhouli, China. Journal of Medical Entomology 2012;49:1283–8.

[93] Xia H, Liu R, Zhao L, et al. Characterization of Ebinur Lake Virus and Its Human Seroprevalence at the China–Kazakhstan Border. Frontiers in Microbiology 2020;10.

[94] Zeng X. [Isolation of Japanese B encephalitis virus from *Culex* (*Barraudius*) *modestus* Ficalbi in Shenyang area (author’s transl)]. Zhonghua Yu Fang Yi Xue Za Zhi [Chinese Journal of Preventive Medicine] 1980;14:209–10.

[95] Becker N, Petrić D, Zgomba M, et al. Mosquitoes and Their Control. 2nd ed. Heidelberg: Springer Nature; 2010.

[96] Sharifi F, Banafshi O, Rasouli A, et al. Biodiversity and Spatial Distribution of Mosquitoes (Diptera: Culicidae) in Kurdistan Province, Western Iran. Journal of Arthropod-Borne Diseases 2023;16:350.

[97] Khoobdel M, Keshavarzi D, Hassan Mossa-Kazemi S, et al. Species diversity of mosquitoes of the Genus *Culex* (Diptera, Culicidae) in the coastal areas of the Persian Gulf. AIMS Public Health 2019;6:99–106.

[98] Adham D, Moradi-Asl E, Vatandoost H, et al. Ecological niche modeling of West Nile virus vector in northwest of Iran. Oman Medical Journal 2019;34:514–20.

[99] Amini M, Hanafi-Bojd AA, Aghapour AA, et al. Larval habitats and species diversity of mosquitoes (Diptera: Culicidae) in West Azerbaijan Province, Northwestern Iran. BMC Ecology 2020;20.

[100] Navidpour S, Vazirianzadeh B, Harbach R, et al. The Identification of culicine mosquitoes in the Shadegan wetland in Southwestern Iran. Journal of Insect Science 2012;12.

[101] Saghafipour A, Abai MR, Farzinnia B, et al. Mosquito (Diptera: Culicidae) fauna of qom province, Iran. Journal of Arthropod-Borne Diseases 2012;6:54–61.

[102] Azari-Hamidian S, Yaghoobi-Ershadi MR, Javadian E, et al. Distribution and ecology of mosquitoes in a focus of dirofilariasis in northwestern Iran, with the first finding of filarial larvae in naturally infected local mosquitoes. Medical and Veterinary Entomology 2009;23:111–21.

[103] Khoshdel-Nezamiha F, Vatandoost H, Azari-Hamidian S, et al. Fauna and larval habitats of Mosquitoes (Diptera: Culicidae) of West Azerbaijan Province, Northwestern Iran. Journal of Arthropod-Borne Diseases 2014;8:163–73.

[104] Hasson RH. Mosquitos’ Species of Diyala Province, Iraq. International Journal of Environment, Agriculture and Biotechnology 2017;2.

[105] *Culex modestus* Ficalbi, 1890 | Walter Reed Biosystematics Unit (WRBU) n.d. https://wrbu.si.edu/vectorspecies/mosquitoes/cx_modestus (accessed August 8, 2023).

[106] Khodzhaeva LF & Issi IV. [A new genus of microsporidans *Cristulospora* gen. n. (Amblyospiridae) with 3 new species from blood-sucking mosquitoes in Uzbekistan]. Parazitologiia 1989;23:140–5.

[107] Pridantseva EA, Lebedeva NI, Shcherban’ ZP, et al. [An evaluation of the possibility of using *Romanomermis iyengari* Welch mermithids for mosquito control in Uzbekistan]. Med Parazitol (Mosk) 1990:15–7.

[108] Arroussi DER, Bouaziz A, & Boudjelida H. Mosquito survey reveals the first record of *Aedes* (Diptera: Culicidae) species in urban area, Annaba district, Northeastern Algeria. Polish Journal of Entomology 2021;90:14–26.
